# Invasive meningococcal disease in older adults: current perspectives and call for action

**DOI:** 10.1007/s41999-024-00969-0

**Published:** 2024-05-06

**Authors:** Catherine Weil-Olivier, Muhamed-Kheir Taha, Sean Leng, Ener Cagri Dinleyici, Paolo Bonanni, Elena Moya, Andreas Leischker, Saber Yezli

**Affiliations:** 1 Université Paris Cité, Paris, France; 2 Institut Pasteur, Invasive Bacterial Infections Unit, National Reference Centre for Meningococci and Haemophilus Influenza, Université Paris Cité, Paris, France; 3 Division of Geriatric Medicine and Gerontology, Department of Medicine, Johns Hopkins University School of Medicine, Johns Hopkins Center on Aging and Immune Remodeling, Baltimore, Maryland USA; 4 Department of Pediatrics, Faculty of Medicine, Eskisehir Osmangazi University, Eskisehir, Türkiye; 5 Department of Health Sciences, University of Florence, Florence, Italy; 6 Europe Regional Coordinator, The Confederation of Meningitis Organizations (CoMO), Madrid, Spain; 7 Working Group “Vaccination”, German Geriatric Society, and Department for Geriatrics, Asklepios Hospital Wandsbek, Hamburg, Germany; 8 Biostatistics, Epidemiology and Scientific Computing Department, King Faisal Specialist Hospital and Research Centre, Riyadh, Saudi Arabia

**Keywords:** Invasive meningococcal disease, Older adults, Increased risk, Immunization, Vaccine equity

## Abstract

**Purpose:**

Invasive meningococcal disease (IMD) is a devastating condition. While most attention is directed towards disease in children and adolescents, IMD poses an important cause of morbidity and mortality in adults ≥60 years. While immunization is a critical component of healthy ageing strategies, meningococcal immunization is not routinely offered to older adults. The aim of this review was to summarize clinical and epidemiological aspects of IMD and available immunization strategies, with a particular focus on disease in older individuals, to emphasize the importance of this rather neglected area.

**Methods:**

An expert working group was established to evaluate clinical and epidemiological data to raise awareness of IMD in older individuals, and develop suggestions to improve the existing burden.

**Results:**

Routine child and adolescent meningococcal immunization has substantially reduced IMD in these targeted populations. Consequently, prevalence and proportion of IMD among those ≥60 years, mostly unvaccinated, is increasing in developed countries (accounting for up to 25% of cases). IMD-related mortality is highest in this age-group, with substantial sequelae in survivors. IMD due to serogroups W and Y is more prevalent among older adults, often with atypical clinical features (pneumonia, gastrointestinal presentations) which may delay timely treatment.

**Conclusions:**

IMD in older adults remains overlooked and greater awareness is required at clinical and societal levels. We encourage clinicians and immunization policy makers to reconsider IMD, with a call for action to remedy existing inequity in older adult access to protective meningococcal immunization.

**Supplementary Information:**

The online version contains supplementary material available at 10.1007/s41999-024-00969-0.

## Key summary points

**Aim:**

Invasive meningococcal disease (IMD) presents a substantial burden in older adults, usually ineligible for protective immunization. We reviewed clinical epidemiology and current immunization policies to highlight current unmet needs in older adults.

**Findings:**

IMD in older adults represents a substantial proportion of the overall disease burden. Atypical presentations are common, often associated with less common serogroups.

**Message:**

While most attention is on disease in younger individuals, there remains a need to increase awareness of IMD in older adults and reconsider immunization policy.

**Supplementary Information:**

The online version contains supplementary material available at 10.1007/s41999-024-00969-0.

## Introduction

Although relatively uncommon, invasive meningococcal disease (IMD) due to *Neisseria meningitidis* remains an important global public health concern [1]. While meningitis and/or septicemia are the most well-known forms of IMD, atypical presentations are increasingly recognized (meningococcal bacteremic pneumonia, acute gastrointestinal upset, septic arthritis) [2, 3]. *N. meningitidis* is usually transmitted by airborne droplets. However, sexual transmission has been reported and *N. meningitidis* can provoke urogenital and anorectal infection, chiefly in men who have sex with men (MSM) [4]. Even with appropriate treatment, case-fatality rates are high, ranging between 4–20% [5]. Serious sequelae e.g., amputations or limb impairment and neurological complications (seizures, hearing loss) are reported in up to 20% of survivors, in whom long-term physical, psychological and social disability and reduced quality of life may be substantial [6].

Global epidemiology shows substantial geographical and temporal variation in the relative importance of specific serogroups in local IMD epidemiology [7]. Most meningococcal infections are due to six serogroups (A, B, C, W, Y and X) [1, 7, 8]. More detailed molecular/genomic analyses allow identification and grouping of specific, often hypervirulent, highly transmissible clonal complexes (cc) spanning different serogroups and with evidence of capsular switching e.g., from serogroup C to W (or B) serogroups [9]. Serogroup C cc11 was associated with much of the past burden of serogroup C disease in developed countries and remains an important cause of IMD outbreaks [9]. W cc11 was associated with the emergence of serogroup W as a globally important IMD causative serogroup [10, 11], and serogroup B cc11 strains are associated with IMD outbreaks [12].

In most developed countries, before any introduction of national immunization strategies, the majority of IMD was due to B, C, W, and Y serogroups, with the greatest incidence in children <5 years of age (in particular infants <12 months) and then in adolescents and young adults aged 15–24 years [1, 13]. To date, these age-groups have been the focus of strategies of immunization programmes in most countries that have implemented vaccines targeting one or more of these specific serogroups [1, 8, 14]. Use of polysaccharide-protein conjugated monovalent MenC and quadrivalent MenACWY vaccines (and more recently protein-based MenB vaccines) has played an important role in contributing to global declines in IMD incidence, and burden (in terms of morbidity and mortality) in children and adolescents [8, 15, 16]. This has led to a shift in IMD age demographics, with increasing case numbers, and a greater proportion of overall cases now involving older adults, evident across much of Europe and in North America [17]. This rather contradicts the conventional thinking that meningococcal disease is a disease of children and younger adults (and by extension, that only these age-groups would benefit from immunization). In reality, up to 25% of the overall IMD burden in many countries is found in older adults [18–20]. Furthermore, as we report later, mortality is higher in this older population, with case fatality rates of 30% reported in adults >75 years of age [5]. Additionally, the frequency of sequelae increases the risk of dependency in older adults.

This constitutes a particular concern. Furthermore, in light of shifting population demographics towards an older ageing population structure in developed countries [21], it may be anticipated that, without any change to existing policies, this burden will increase. There is a need to raise awareness of IMD, within and across medical specialties involved in the care of older adults, in order to communicate greater understanding and improve clinical outcomes. In addition, the present approach in immunization programs, whereby routine adult meningococcal immunization is not currently available merits reconsideration.

Against this background, an international multidisciplinary expert working group (EWG) was formed to evaluate the existing knowledge base of meningococcal disease in older adults. For the purposes of the present paper, we consider this in the context of individuals aged ≥60 years, although this in itself spans a broad age-group and includes many that can be considered middle aged adults. The purpose was to identify knowledge gaps and develop proposals for future initiatives to address such limitations (See Supplementary Material and Supplementary Fig. 1). The proceedings from these initial meetings are reflected in this paper.

## Methods

### Search strategy and selection criteria

We searched PubMed using a range of free-text and MeSH search terms in various combinations to identify relevant publications reporting meningococcal infection epidemiology, disease burden, outcomes and immunization policy in older adults. This was supplemented by reviewing the reference lists of relevant publications from identified papers. Although comprehensive, this was not formally systematic, with no specific selection criteria or date limitations, searching through May 2023. Additional data were sourced from public databases reporting national surveillance data, e.g., the European Centre for Disease Prevention and Control (ECDC) and the Centers for Disease Control and Prevention (CDC).

### Healthy ageing and the value of adult immunization

Shifting population demographics, with an increasingly ageing population [21, 22], places a premium on optimising older adults’ health and quality of life, whilst maintaining an individual’s personal capabilities and autonomy within the framework of broader ‘healthy ageing’ [23]. The risk of infectious disease, including vaccine-preventable disease in older adults is influenced by age-associated senescent remodeling in humoral and cell-mediated immunity (immunosenescence) [24]. Vaccine-preventable diseases pose a substantial disease and economic burden in older adults [25], and so immunization is a critical component of any healthy ageing strategy [26, 27]. Indeed, whilst immunization policies and initiatives in most countries are age-based with their principal focus upon child and adolescent populations, a lifelong vaccination approach has been increasingly adopted [28]. In this approach, individuals across all-age-groups are offered all appropriate vaccinations (including catch-up vaccination when prior scheduled vaccination was missed, and regular boosters). For older adults, although it varies across specific countries, this approach includes immunization against influenza, pneumococcal infection, pertussis and herpes zoster, in addition to continued SARS-CoV-2 vaccination, and may extend towards more recently developed vaccines such as those against respiratory syncytial virus (RSV). While vaccination for older adults is embedded within some countries’ immunization programs, e.g., the United States (US), France, and Italy [29], broader global implementation of this approach is limited, in part due to perceived financial barriers and other priorities for national vaccination strategies [30]. Even when fully publicly funded programs are in place, disparities in older adult vaccination uptake are apparent, with lower uptake of recommended influenza and pneumococcal vaccines in older adults associated with racial/ethnic factors, income and educational status [31]. Reduced awareness regarding recommendations and benefits for immunization from healthcare providers, and concerns about costs, all play a role in lower vaccine uptake; misinformation is also a factor [32]. This conflicts with the recognized benefits of immunization in reducing morbidity and mortality due to vaccine-preventable disease. There is a wealth of supportive evidence emerging for broader health gains with adult vaccination, notably beneficial effects of influenza immunization in reducing mortality and cardiovascular events in high-risk populations, including older adults [33]. Strategies to improve vaccine uptake, including efforts to alleviate any allied vaccine hesitancy, ideally emphasising vaccine benefits within the broader preventive and supportive ‘healthy ageing’ framework, remain a critical component of public health [34].

Prioritising vaccination of the older and most vulnerable older adult population was a successful strategy when addressing the Covid-19 pandemic, and calls to maintain this approach in ongoing broader older adult immunization policy have been made [27]. The pandemic also generated tremendous societal dialogue on the place of older adults within society, where an attitude of ageism, in which older individuals are considered equally frail, lacking in autonomy and with little contribution to society was apparent [35]. This still remains in some quarters, and ignores the often substantial economic contributions of older adults (and even greater social importance within the family and larger community) [36]. Regardless of any net economic gains or burdens, simple fairness demands social and health equity for older adults to address disparities and maintain individual autonomy. This includes access to available vaccines against important vaccine-preventable conditions in older adults. As we outline below, this could and perhaps should include broader publicly funded access to meningococcal immunization.

## Challenges of IMD in older adults

### Clinical aspects

**Fig. 1:**
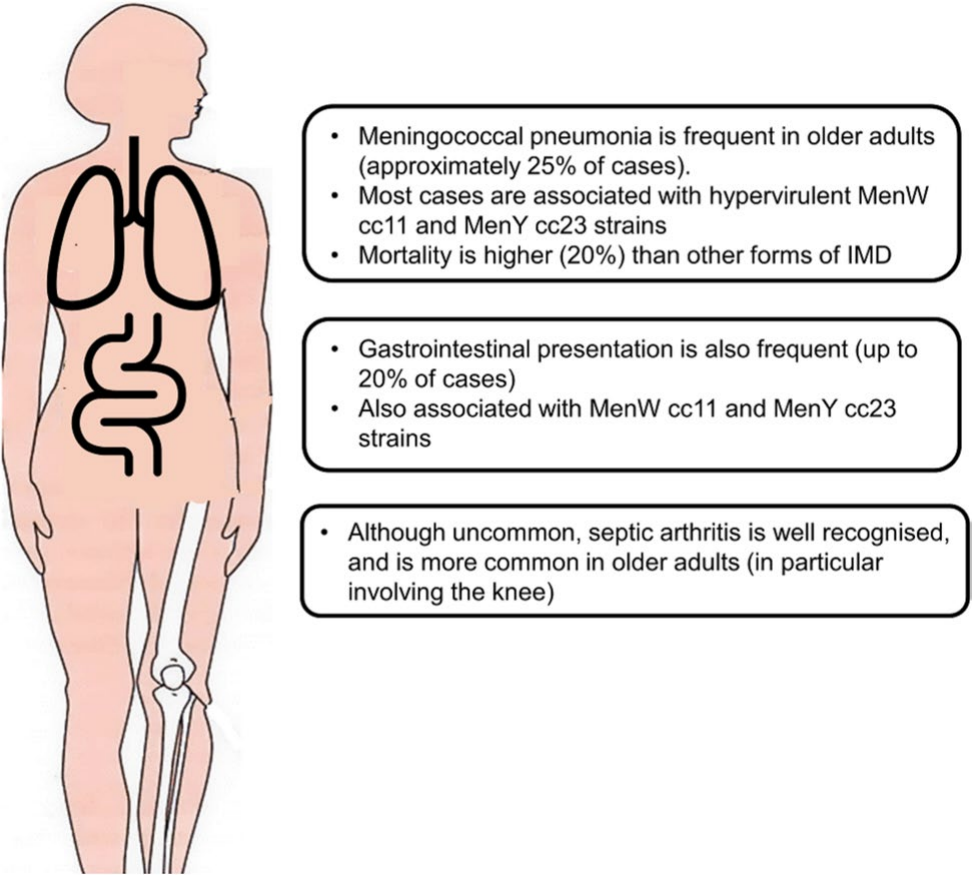
Atypical presentations of meningococcal infections in older adults

Clinical forms of IMD in older adults may differ from those in other age groups. Atypical presentations are far more common in older adults, particularly bacteremic pneumonia, and gastrointestinal presentations, preceding or concurrent with overt meningitis or sepsis, and also septic arthritis [2, 3]. An illustrative schematic is shown in Fig. 1. Studies in several countries clearly show this over-representation and its association with specific hypervirulent W cc11 and Y cc23 strains [2, 3, 37, 38]. In the UK, most meningococcal pneumonia is associated with serogroup Y cc23, and pneumonia has a higher case fatality rate than other clinical forms (19% vs. 17% seen with septicemia) [3]. In France, case fatality in those aged ≥60 years is 20% compared to an overall case fatality rate of 10%, in particular due to serogroups W and Y (17.9% and 22.5% respectively) [39].

Even with more classical presentations, IMD in older adults carries a generally lower index of suspicion; with atypical presentations, IMD is rarely considered in the initial differential diagnosis. Furthermore, older adults with IMD may present to, and be cared for by a wide range of clinical specialists (primary care and emergency physicians, pulmonologists and gastroenterologists etc., as well as geriatric specialists), where there may be limited experience and generally low awareness of IMD in older individuals. This indicates the need for greater education of healthcare professionals, allied with standardized case definitions and diagnostic protocols (to capture all IMD including atypical presentations) to ensure more precise estimation of disease burden.

### Risk factors for IMD in older adults

Conventional risk factors for IMD are well recognized, spanning medical comorbidities (chiefly immunodeficiency) and social factors [40, 41]. In addition to these general risk factors, several risk factors are more specifically encountered among older adults. The CDC considers older age per se (≥65 years) a risk factor [42]. Common medical comorbidities, more prevalent in older individuals e.g., diabetes mellitus, chronic pulmonary and renal disease, are also reported to increase the risk [41, 43], and underlying respiratory comorbidities are commonly reported in older adults with meningococcal pneumonia [3]. Overall comorbidity burden e.g., higher Charlson comorbidity index (CCI) also confers greater IMD risk, and also greater risk of sequelae [43].

Social crowding is strongly associated with risk across all age-groups [44]. This includes nursing home residents, where limited IMD disease clusters in older residents are reported (chiefly MenW cc11 disease, presenting with pneumonia or non-specific respiratory symptoms) [45]. Attendance at mass gatherings, notably Hajj and Umrah pilgrimages to the Kingdom of Saudi Arabia, is a well-recognized risk factor, and of direct relevance to older individuals as approximately 20% of pilgrims are ≥60 years of age [46]. Prior IMD outbreaks associated with Hajj and Umrah events include a major international serogroup A outbreak in 1987, and localized serogroup A outbreaks in 1988, 1992 and 1997 [46]. These were followed by substantial Hajj-related international outbreaks in 2000 and 2001, predominantly due to serogroup W, associated with substantial mortality, especially in the older pilgrim population (with a case fatality rate in those aged >45 years of 32.6%) [47, 48].

## Meningococcal immunization

A broad range of meningococcal vaccines are available (see additional details in Supplementary material). Four quadrivalent conjugate ACWY vaccines are in widespread use, utilising differing carrier proteins (Table 1) [49, 50]. Protective antibody responses against vaccine target serogroups are observed across all age-groups, with robust seroprotective responses observed in adults ≥56 years of age [49, 50]. Conjugate quadrivalent vaccines are known to reduce *N. meningitidis* colonization and carriage limiting thus the circulation of the bacteria and providing indirect protection to unvaccinated individuals, although this is observed chiefly in adolescents and young adults, and the extent towards older adults remains less clear [51]. Two protein-based MenB vaccines are also available, targeting subcapsular protein antigens expressed by pathogenic MenB strains [1, 49, 52]. While these induce seroprotective antibody responses and reduce serogroup B disease, unlike conjugated ACWY vaccines, there is no evidence for reduction in serogroup B carriage and any broader indirect effect [52].

**Table 1:** Licensed conjugated quadrivalent (serogroups A, C, W, Y) and meningococcal B vaccines

Region	Conjugated Quadrivalent vaccine				Men B vaccine	
	MenACWY-CRM (Menveo^®^, GSK)	MenACWY-TT (Nimenrix^®^, Pfizer)	MenACWY-TT (MenQuadfi^®^, Sanofi)	MenACWY-DT (Menactra^®^, Sanofi)	4CMenB (Bexsero^®^, GSK)	MenB-FHbp (Trumenba^®^, Pfizer)
Europe	≥ 2 years	≥ 6 weeks	≥ 12 months	–	≥ 2 months	≥ 10 years
United States	2 months–55 years	–	≥ 2 years		10–25 years	10–25 years
Canada	12 months–55 years	12 months–55 years	≥ 12 months	9 months–55 years	2 months–25 years	10–25 years
Australia	12 months–55 years	12 months–55 years	≥ 12 months	9 months–55 years*	≥ 2 months	≥ 10 years

Illustrative availability and age-based approvals [49, 50, 53]

*4CMenB* 4 component meningococcal serogroup B vaccine, *CRM* Corynebacterium diphtheriae C7 cross-reacting material 197, *MenB-FHbp* bivalent factor H binding protein meningococcal serogroup B vaccine, *TT* tetanus toxoid

*In Australia, Menactra^®^ is being replaced by MenQuadfi^®^

Differences in meningococcal vaccine licensing and approval in different age-groups exist. Only one quadrivalent ACWY vaccine has broad global approval (i.e., in Europe, North America, and Australia, and with no upper age restrictions) [50]. Similarly, one of the two MenB vaccines is licensed with no upper age restrictions (and only within Europe and Australia) [53]. Most countries offer infant, toddler and/or adolescent meningococcal immunization, although specific vaccines and target age-groups vary widely across countries, as comprehensively reviewed by Taha et al. [8]. An illustrative schematic is shown in Supplementary Fig. 2 with explanatory text in the Supplementary material. However, routine meningococcal immunization in adults is not recommended in any national programs; only those adults considered at high-risk. This usually encompasses those at greater risk due to immunological impairment (including people living with HIV), living in specific high-risk environments (e.g., college dormitory students, military recruits), and MSM. For these individuals, quadrivalent and MenB vaccines are offered (even in those countries without established child/adolescent programs). Indeed, these were often in place prior to age-based recommendations. Specific eligibility criteria may vary, e.g., HIV infection is not a specific indication in France [41, 53]. Most countries recommend vaccination of close contacts of affected individuals (“index cases”), although such policies also vary in different countries. However, despite being recommended in high-risk adults, reported uptake of MenACWY and MenB vaccines is low in these groups (<30%) [54]. One exception to less restricted adult meningococcal immunization is the policy implemented for all Hajj/Umrah pilgrims entering Saudi Arabia, where adult MenACWY immunization has been mandatory since 2001 [46, 47]. Although either polysaccharide or conjugated quadrivalent vaccines can be used, conjugated MenACWY vaccines are preferred [46, 47]. However, many pilgrims, particularly those travelling from low- and middle-income countries, receive the polysaccharide vaccine, and indeed despite being mandatory, some report no recent MenACWY vaccination [55]. MenB immunization is not currently required for pilgrims.

## The changing epidemiology of IMD

Temporal shifts, cyclic epidemiolocal patterns, immunization strategies, and the spread of hypervirulent strains have shaped the current IMD landscape [7, 9]. The introduction of childhood and adolescent in national routine immunization programs, utilising conjugated monovalent MenC and MenA, quadrivalent MenACWY and more recent protein-based MenB vaccines, has led to a substantial decline in IMD in target age-groups [1, 8, 49]. The importance and primacy of childhood and adolescent immunization in national programs has been emphasized by the success achieved in strongly reducing IMD incidence rates in these age groups [1, 7] (Supplementary material and Supplementary Fig. 3). In adults, MenACWY vaccination for Hajj and Umrah pilgrims and for residents of the Holy cities, with chemoprophylaxis for pilgrims from Sub-Saharan Africa, has also had great success, with no documented outbreaks since 2001 [46].

**Fig. 2:**
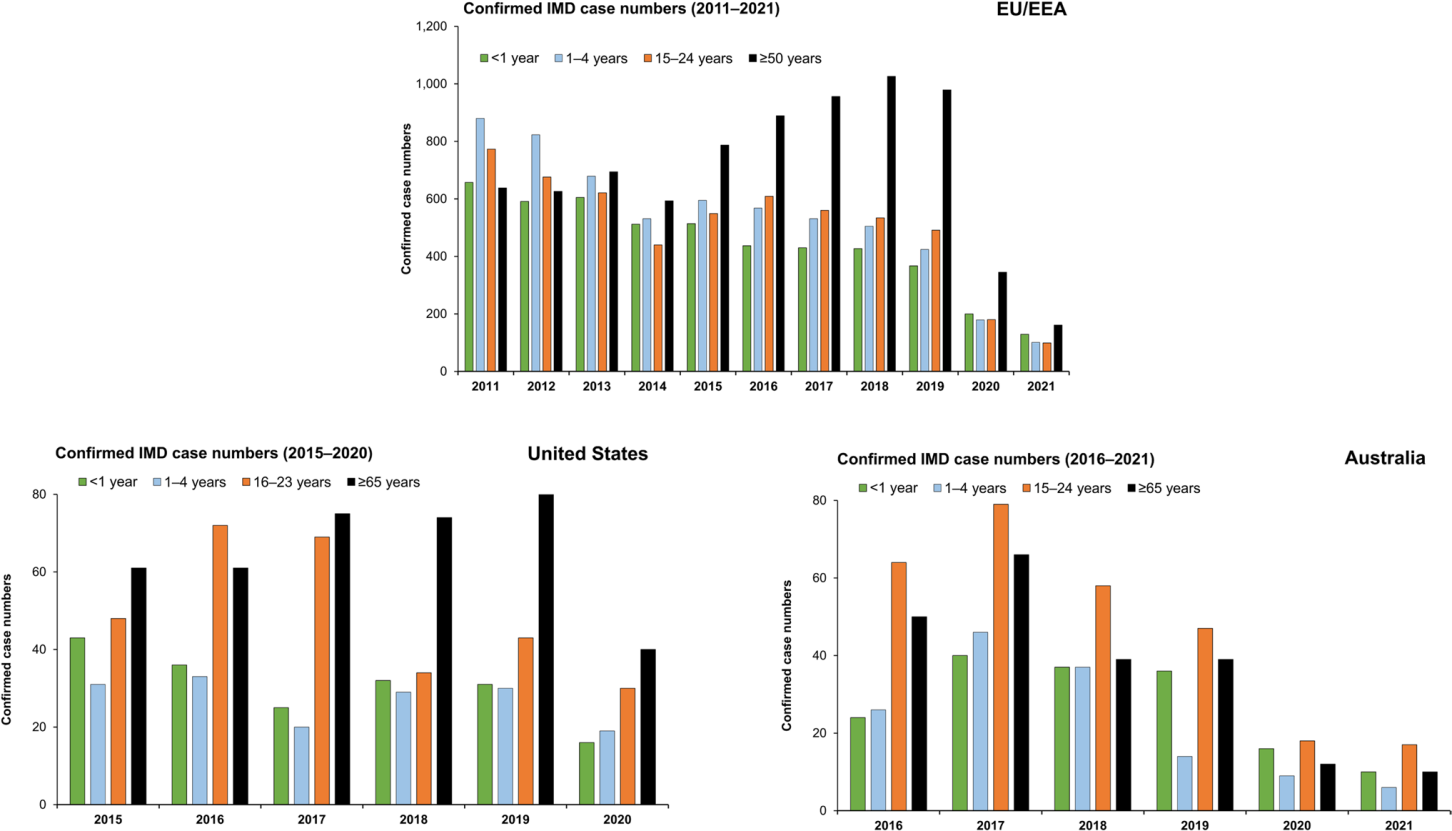
Distribution of invasive meningococcal disease case burden in Europe, the United States and Australia in select age-strata**.** European data sourced from the ECDC Surveillance Atlas of Infectious Diseases tool [18]. Data for the United States as reported by the CDC [19]. Australian data sourced from national surveillance reports [20]

**Fig. 3:**
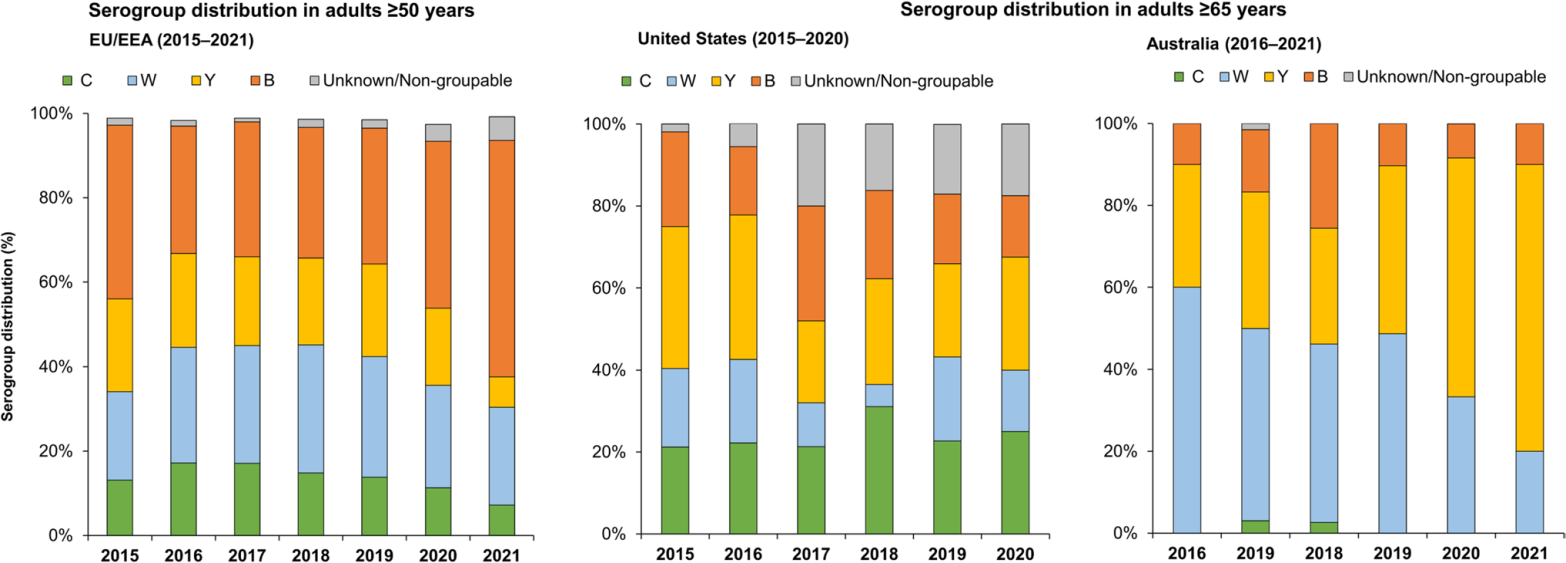
IMD serogroup distribution in older adults (≥50 years) in Europe and those ≥65 years in the United States and Australia. European data sourced from the ECDC Surveillance Atlas of Infectious Diseases tool [18]. Data for the United States as reported by the CDC [19]. Australian data sourced from national surveillance reports [20]

**Fig. 4:**
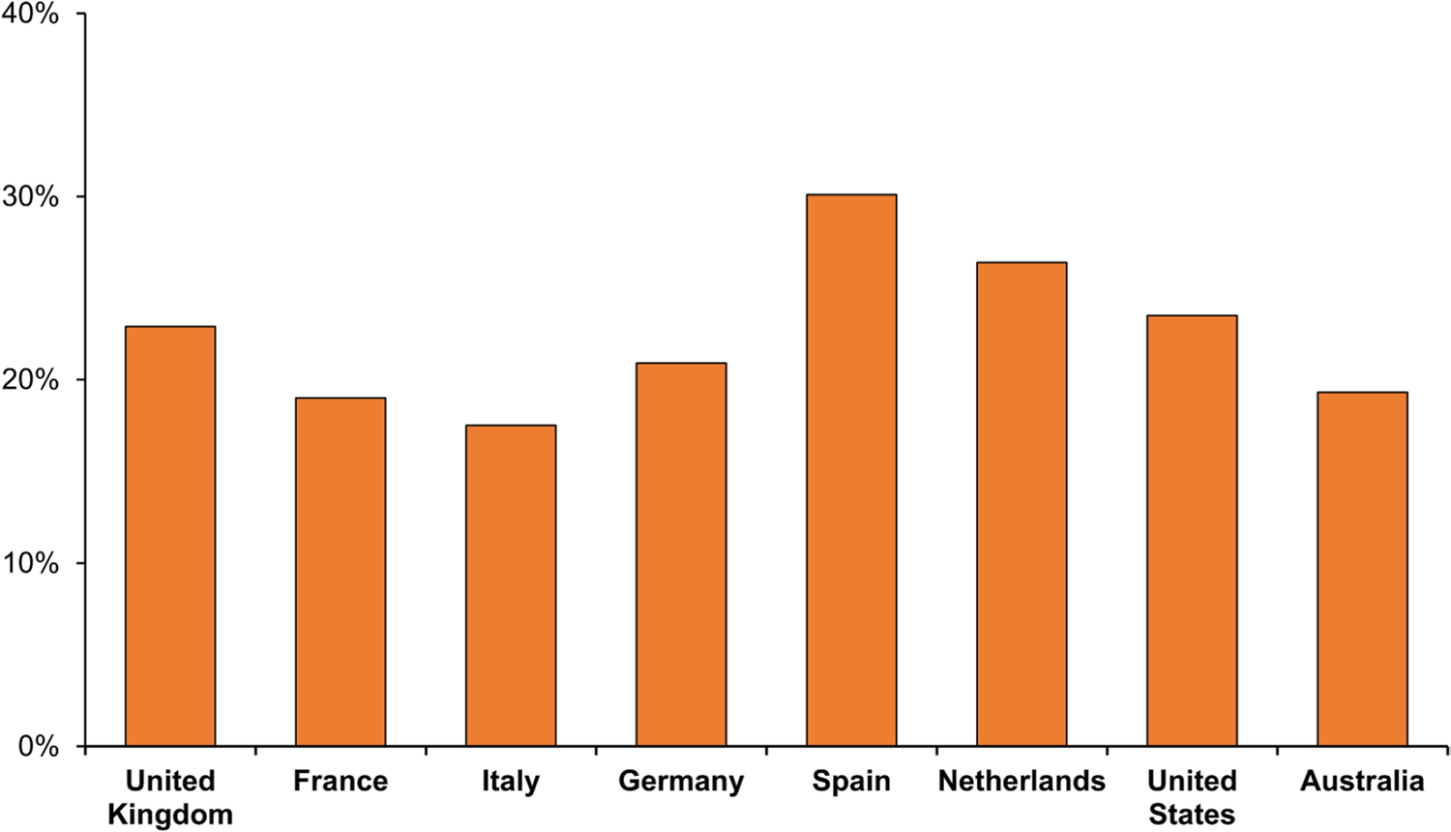
The proportion of all IMD cases which involved those aged ≥65 years in select countries in 2019. European data sourced from the ECDC Surveillance Atlas of Infectious Diseases tool [18]. Data for the United States as reported by the CDC [19]. Australian data sourced from national surveillance reports [20]

In Europe, declines in disease in infants, young children and adolescents and young adults have been observed since 2011, with a shift in IMD age demographics, whereby case numbers in those aged ≥50 years of age have increased almost two-fold since 2014 [18] (Fig. 2). In 2019, a total of 979 IMD cases in the EU occurred in those aged ≥50 years, which accounted for 33.3% of all IMD; with 20% of all cases involving those ≥65 years [18]. In the most recent years prior to the Covid-19 pandemic a significant proportion of all cases in adults ≥50 years were due to serogroups W (21–30%) and Y (20–22%) (Fig. 3). Similar patterns are seen when appraising surveillance data in specific European countries (Fig. 4 and Supplementary Fig. 4). In France, between 2012–2017, 19.2% of all IMD cases involved adults ≥60 years [41]. In the United Kingdom (UK), 12.2% of all IMD cases between 2008–2017 involved adults ≥50 years (and accounted for over 20% of cases in 2016 and 2017) [43]. Appraising ECDC data for 2019, 31% of all cases in France involved patients aged ≥50 years (and 19% in those ≥65 years); in the UK over 36% of all cases were in those ≥50 years, and 22.9% in those ≥65 years [18]. In this same year the proportion of cases in those ≥65 years ranged from 17.5% in Italy to 30.1% in Spain (Fig. 4). Across all these countries, serogroup W and Y disease was highly prevalent in older adults [18].

These patterns are also observed in North America and elsewhere. In the US, CDC data indicates that disease in adults ≥65 years of age accounted for 20.1% of all IMD cases between 2015–2019 (359 cases in this age-group) and 23.5% in 2019 [19]. IMD due to B, C, W, and Y serogroups was observed in relatively similar proportions (Figs. 2 and 3). In Canada, between 2012–2019, 24.9% of all cases occurred in older adults ≥60 years, mainly due to Y, W and B serogroups [56]. In Australia, 17.7% of all IMD cases reported between 2016 –2019 involved adults ≥65 years of age (194 cases) [20], with the majority caused by serogroups W and Y (50.0% and 33.0% respectively) (Fig. 3).

The widespread social and individual restrictions implemented to restrict viral transmission and mitigate the impact of the Covid-19 pandemic was accompanied by often substantial declines in a wide range of respiratory transmissible infections, most notably pneumococcal and *H. influenzae* infection, but also for IMD [57, 58]. Sharp declines in IMD case numbers in 2020 were observed in most countries, and across most age-groups [18–20, 58–61] (Fig. 2). However, despite reduced case numbers, IMD in older adults remained frequent, often with atypical (respiratory) presentations [2]. There is, as yet, limited data on IMD in the post-pandemic setting. Emerging, often preliminary data indicate that while case numbers remain lower than those observed prior to the pandemic in all countries, some resurgence in 2022 is apparent, and a substantial proportion affects older adults. In the UK, although IMD burden is still lower than that observed in 2019/20, increases in case numbers for 2022 compared to 2021 are reported [62]. Although these are most apparent in younger individuals aged 15–19 years (predominantly serogroup B), 9% of all IMD cases in 2022 were in those aged 45–64 years and a further 10% in those aged ≥65 years (chiefly due to B and W serogroups) [62]. In France, IMD case numbers have increased from mid-2021 onwards, particularly in the latter half of 2022 [63]. While again this is most notable in 15–24-year-olds, and which indeed also exceeded pre-pandemic levels, a marked rebound in IMD due to Y and W serogroups in adults was also reported (and in particular in those aged ≥65 years, which accounted for 17% of all IMD cases) [63]. In the US, preliminary data indicates some increase in cases in 2022 compared to that reported for 2020/21, including IMD due to serogroups C, W and Y in older adults [64]. In Australia, while case numbers remain lower than pre-pandemic levels, 30% of cases in 2021 involved adults aged ≥45 years, and 15% in those ≥65 years (predominantly serogroup Y which accounted for 70%) [20]. Although more complete, definitive data are required to evaluate these patterns in the post-Covid setting, it would seem that disease in older adults continues to be an important factor in the overall IMD burden in many developed countries.

## The impact of IMD in older adults

### IMD case-fatality rates in older adults

**Fig. 5:**
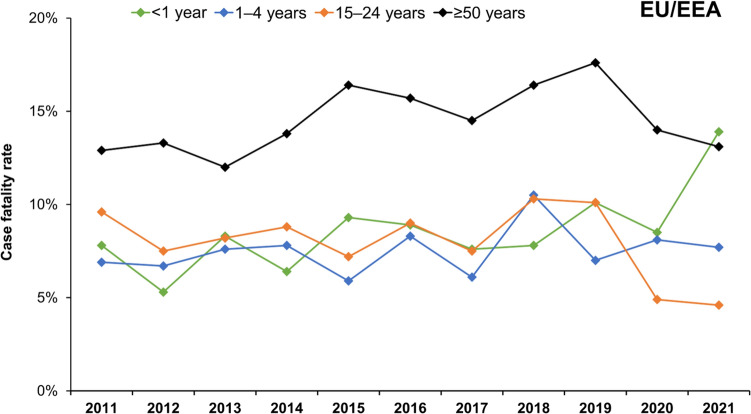
Invasive meningococcal disease case fatality rates in Europe (2011–2021). Data sourced from the ECDC Surveillance Atlas of Infectious Diseases tool [18]

Mortality is substantially higher in older adults [5]. One study evaluating IMD in the UK over the 2008–2017 period reported a case fatality rate of 21.9% in those aged ≥50 years [43], while another UK analysis (over 2008–2015) reported fatality rates of up to 22.8% in those aged ≥65 years [65]. Data from the ECDC show case fatality rates of up to 17.6% in individuals ≥50 years in 2019 (Fig. 5), when 49.4% of all IMD deaths across Europe involved individuals ≥50 years (with 29.9% in those aged ≥65 years) [18]. Rates in specific countries are even higher; in France, for 2019 the case fatality rate in individuals ≥50 years was 26% [18] (Supplementary Fig. S5). While most deaths occur in the acute phase, later mortality does occur (and mortality may be underestimated). Indeed, there is increasing interest in the concept of earlier mortality in adults surviving IMD [66]. These IMD mortality rates may exceed those reported for the more common vaccine-preventable disease involving older adults. For example, for adults ≥65 years hospitalized with pneumococcal pneumonia, fatality rates of between 10–12% are reported [67].

### IMD sequelae in older adults

Survivors are at risk of developing a wide range of long-term (and often life-long) physical, neurological and psychological sequelae of differing severity [6]. Physical sequelae range from skin necrosis and subsequent scarring requiring skin grafting to limb amputations (following septicemia). Patients may also develop or aggravate pre-existing chronic cardiovascular and renal conditions [6, 68]. Neurological impairment is common; mild to severe hearing loss is reported in 3–8% of adults [6]. Data on other neurological sequelae frequently reported in younger children (epilepsy/seizures, cognitive impairment) are more limited for older survivors. Psychological sequelae (including depressive and anxiety disorders) may also be overlooked. While these (and other psychosocial/behavioural problems e.g., post-traumatic stress disorder and attention deficit hyperactivity disorder) are apparent in childhood IMD survivors [6], there are no data for older adults.

There remains a need to better characterize the impact of IMD on the psychological wellbeing and broader quality of life in older adults, and also to quantify the potential loss of autonomy (“dependency”) following severe infectious disease episodes, including IMD. As those of working age may take early retirement and/or invalidity benefits following IMD [66, 68], some impact may be expected. Similarly, while spill-over effects are increasingly recognized, with often prolonged psychological impact on the parents and wider family of affected children [69], there are little data for this in the context of older adult survivors, and the impact on their partners, adult offspring and others involved in providing practical and emotional support.

### Economic aspects

Vaccine-preventable disease in older adults poses a substantial clinical and economic burden [25]. Data are limited on the economic impact of IMD in older adults, and there is a need to quantify this better [70]. However, these may be substantial, and IMD costs may be higher than other forms of bacterial meningitis [68]. Direct medical costs are generally highest in older patients [68, 71]. Cost estimates for the acute disease phase of IMD in patients ≥60 years of between €10,585–16,132 are reported for Germany [72] and between €13,365–14,965 in France, where the index hospitalization costs increases significantly with age, and is highest among those ≥60 years [68]. Management of complications during the initial phase and of subsequent sequelae carries additional direct costs [68]. While data are rather limited for complication costs in older adults (and lifetime horizon expenditure), annual costs of €10,000 are reported [68]. Indirect societal costs (patient and caregiver productivity losses, social care packages etc.) add further to the broader economic burden of IMD in older adults (although there are scanty data for this).

Together, the high mortality, with the substantial sequelae in survivors, along with high direct and indirect costs, all point towards a significant burden of IMD in older adults, beyond that which may be expected from a more limited perspective of prevalence alone (which in itself may be underestimated). This said, although IMD in older adults ≥60 years constitutes as much as 25% of the overall IMD burden, taking only into account the low incidence rate in older adults means that any broader policy may not be cost-effective under existing cost-effectiveness frameworks*.* One recent economic evaluation in Italy examining use of adolescent MenB vaccination, reported that, even with a relatively small number of IMD cases in the adolescent population, a universal MenB vaccination policy would be cost-effective. In this, much of the benefits are due to substantial direct and indirect cost savings associated with sequelae, incurred by a relatively small number of survivors [73]. This points towards high healthcare and societal costs of sequelae, which need also apply to older adults.

## The importance of meningococcal vaccination in the older adult population

At present, only older adults considered at high risk due to immunological impairment are eligible for meningococcal immunization, and the present focus on predominantly pediatric and adolescent immunization policy recommendations means that many adults remain at continued risk. In light of the high prevalence of IMD in older adults and their consequences, we would maintain that current policies and their implementation remain inadequate to address the present imbalance.

As a starting point, in established at-risk adults, reported uptake of meningococcal vaccines is low and must be improved [54]. In addition, for pilgrims travelling to Saudi Arabia, greater use of conjugate MenACWY vaccines would be welcome [46]. However, we would go further. Meningococcal immunization policies are constantly evolving in response to local/national epidemiologic changes, risk factors and new vaccine availability. It can be argued that ageing itself is an additional risk factor for IMD, their debilitating sequelae and consequences (loss of independence; decreased quality of life). While the processes underpinning *N. meningitidis* acquisition and subsequent development of IMD in older adults are poorly understood, it may be that immunosenescence plays a role. The greater risk of IMD in immunocompromised individuals provides some, albeit indirect evidence for this [41]. The high prevalence of disease in older adults also provides some support for older age as a specific risk factor. A more harmonized approach to meningococcal immunization strategies that includes older adults would seem more equitable and more consistent. While it could be expected that there is sufficient indirect ‘herd’ protection of the older population provided by adolescent MenACWY vaccination, there is no clear evidence of this so far, with the greatest benefit of this seen in adolescents [51].

Our concern is that despite considerable progress in combating IMD in younger individuals through immunization, older adults are in danger of being left behind in this endeavor. In their global “Defeating meningitis by 2030 roadmap” the WHO calls for equitable access to protective vaccines, with goals of reducing vaccine-preventable bacterial meningitis cases by 50% and deaths by 70% compared to 2015 levels [74]. While this chiefly relates to childhood vaccine access (and in less-developed countries), it may be argued that similar considerations also apply to older vulnerable populations. Clearly any expansion of publicly funded meningococcal immunization policy to include older adults would involve substantial implementation costs. This presents challenges to public health agencies, where immunization programs and wider healthcare funding are under increasing pressure. As others have reported, there is need for alternative more equitable approaches to valuing vaccination of older adults, that accommodates broader, non-economic benefits (e.g., family and societal contributions, and spillover effects) to inform and support such decision-making [75, 76].

## Calls to action

The burden of IMD in older adults we describe would seem to demand greater attention be paid to this issue. There remains an urgent need to raise awareness amongst all healthcare professionals (and the general public) of this. Atypical presentations can incur diagnostic and therapeutic delay and adversely affect outcomes, and may impact the accuracy of IMD surveillance and subsequent epidemiological reports. We call for a broader research agenda into IMD in older adults. Our own initiatives in development include research into improved protocols to assist diagnosis and treatment, and we encourage others to pursue studies to expand the knowledge base and bridge existing knowledge gaps. While most systems report age-demographics, these are usually within rather broad strata, e.g., ≥50 years or ≥65 years. There remains a need to report such data in more granular detail to better understand burden and impact on older age-groups e.g., those over 80 years of age. In turn this may help determine which older individuals are at greatest risk (and may benefit most from vaccination). A clearer understanding of any indirect ‘herd’ protection for older adults gained from existing immunization programs is a further research-gap, while additional studies investigating the impact of older adult vaccination on carriage rates and any potential broader indirect protective effect on decreased carriage in children are welcome.

Decision-making requires a greater understanding of the cost burden of IMD in older adults, and of the broader impact on survivors in terms of sequelae, loss of autonomy and poorer quality of life. Studies examining these aspects are welcome. In addition, increased education on available vaccines, regardless of reimbursement considerations, is essential to inform patient choices.

### Supplementary Information

Below is the link to the electronic supplementary material.

Supplementary file 1 (DOCX 785 kb)

## Data Availability

The datasets reported in this review are publicly available.
