# Supplementary material for: Invasive meningococcal disease in older adults: current perspectives and call for action
Source: Eur Geriatr Med. 2024 May 6;15(3):729–41. doi: 10.1007/s41999-024-00969-0 (PMC11329530; doi:10.1007/s41999-024-00969-0)
Supplement: Supplementary file 1 — (DOCX 785 kb) [file 41999_2024_969_MOESM1_ESM.docx]

Supplementary Material

**Invasive meningococcal disease in older adults – Current perspectives and call for action**

Catherine Weil-Olivier,^1^ Muhamed-Kheir Taha,^1,2^ Sean Leng,^3^ Ener Dinleyici,^4^ Paolo Bonanni,^5^ Elena Moya,^6^ Andreas Leischker,^7^ Saber Yezli^8^

**Affiliations**

^1^ Université Paris Cité, Paris, France

^2^ Institut Pasteur, Invasive Bacterial Infections Unit, National Reference Centre for Meningococci and Haemophilus Influenza, Université Paris Cité, Paris, France

^3^ Division of Geriatric Medicine and Gerontology, Department of Medicine, Johns Hopkins University School of Medicine, Johns Hopkins Center on Aging and Immune Remodeling, Baltimore, Maryland, USA

^4^ Department of Pediatrics, Eskisehir Osmangazi University Faculty of Medicine, Eskisehir, Turkey

^5^ Department of Health Sciences, University of Florence, Florence, Italy

^6^ Europe Regional Coordinator, The Confederation of Meningitis Organisations (CoMO), Madrid, Spain

^7^ Working Group “Vaccination“, German Geriatric Society and Department for Geriatrics, Asklepios Hospital Wandsbek, Hamburg, Germany

^8^ Biostatistics, Epidemiology and Scientific Computing Department, King Faisal Specialist Hospital and Research Centre, Riyadh, Saudi Arabia

**Contents**

[**Methodology** 2](#_Toc153536961)

[Supplementary Fig. 1 EWG approach 3](#_Toc153536962)

[**Meningococcal immunisation** 4](#_Toc153536963)

[**Programmatic aspects** 4](#_Toc153536964)

[**IMD incidence** 6](#_Toc153536965)

[**References for Supplementary Material** 9](#_Toc153536966)

**Methodology**

The expert working group (EWG) comprised seven members, each specialists in infectious disease epidemiology and clinical management or within broader areas of immunization policy from across Europe, the United States and the Middle East. An eight member was a patient advocate (from the Confederation of Meningitis Organisations). A sequence of virtual online meetings was held in May 2022. The first encompassed a general broad discussion on the available knowledge of IMD epidemiology and outcomes in older adults and existing knowledge gaps. The second meeting focused specifically on developing initiatives to address identified knowledge-gaps in knowledge and awareness (Supplementary Fig. 1). Following these, the EWG then convened in-person on October 5^th^ 2022 to develop an action plan to develop a series of manuscripts reporting on their findings and status of ongoing initiatives. For the present manuscript, the authors convened on-line (27^th^ January 2023) to discuss the content of the present manuscript, with a focus on how best to raise awareness.

The logistics for the EWG meetings and subsequent activities were supported by Sanofi. The sponsor had no influence on the recommendations developed from this process. The proceedings from these initial meetings are reflected in this present paper.

**Supplementary Fig. 1** EWG approach


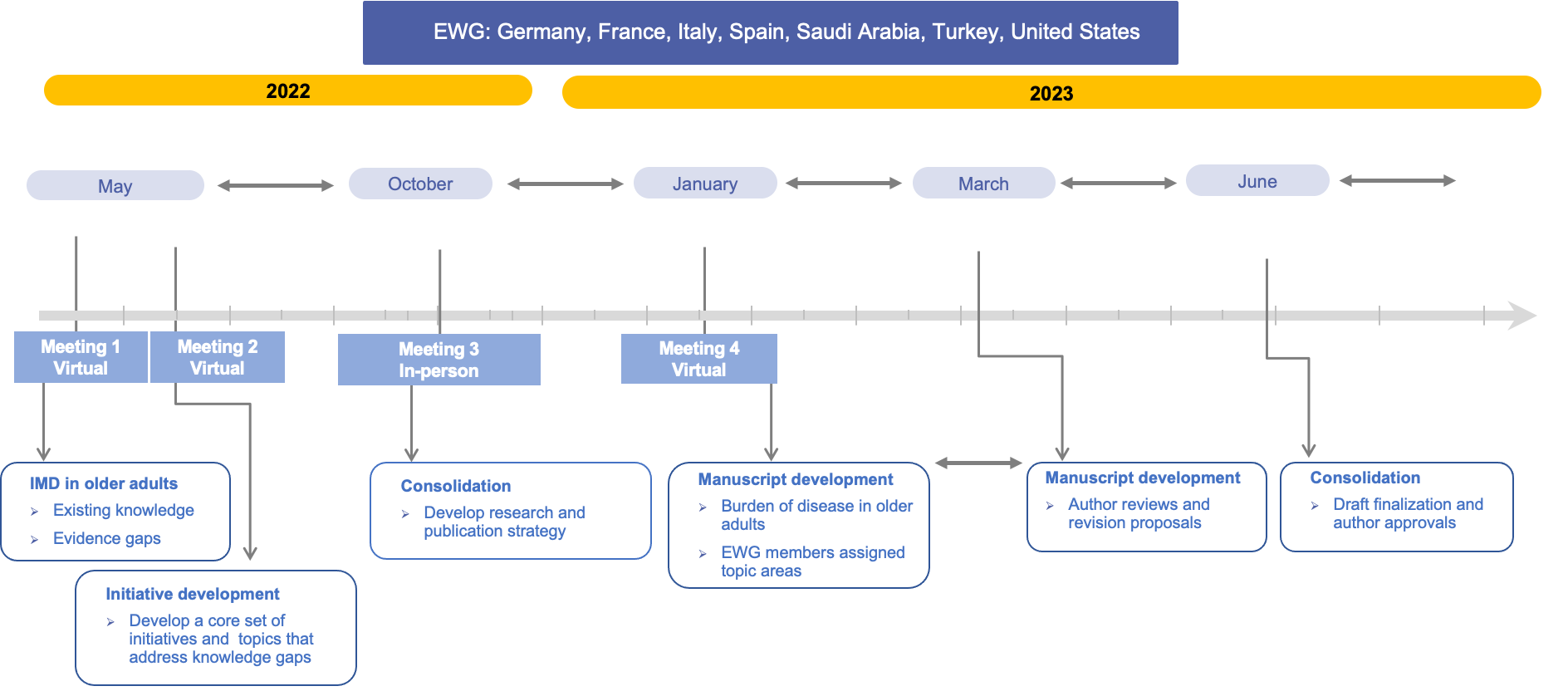


**Meningococcal immunization**

A broad range of meningococcal vaccines are available. While older unconjugated polysaccharide vaccines remain in use in some settings (e.g., China and in some countries for use by Hajj/Umrah pilgrims), countries with established programmes use protein-conjugated vaccines in monovalent and quadrivalent forms (MenA, MenC, and MenACWY vaccines). Conjugated vaccines induce T-cell dependent responses, generating greater immune responses and more persistent antibody responses and improved responses to booster doses [[1](#_ENREF_1),[2](#_ENREF_2)]. In addition, conjugated vaccines , in particular when used in adolescent immunization (in whom carriage rates are highest) may reduce *N. meningitidis* acquisition and onward transmission, providing indirect ‘herd’ protection across the broader population, including those age-groups not targeted in routine immunization programmes [[2](#_ENREF_2),[3](#_ENREF_3)].

Robust evidence exists for the indirect protection against disease due to C and A serogroups with monovalent vaccines [[4](#_ENREF_4),[5](#_ENREF_5)]. While a broader effect of indirect protection against other serogroups with MenACWY vaccines has been questioned [[4](#_ENREF_4)], there exists sound evidence from studies from the UK following introduction of adolescent MenACWY immunization in 2015 indicating reduced carriage of C, W and Y serogroups (and important clonal complexes e.g., serogroup W cc11Y cc23) and crucially, reductions in invasive meningococcal disease (IMD) due to W and Y serogroups, beyond that expected for any direct protection alone [[6](#_ENREF_6),[7](#_ENREF_7)]. Indeed, the estimated indirect protection realized with the MenACWY programme was greater than the estimated direct effect [[7](#_ENREF_7)]. It should be noted, however, that these effects were observed chiefly in adolescents and young adults, and the extent of herd-protection towards older adults remains less clear.

The introduction of widespread MenACWY vaccination strategies for Hajj and Umrah pilgrims and for residents of the Holy cities, and chemoprophylaxis for pilgrims from Sub-Saharan Africa, has had great success, with no documented outbreaks since 2001[[8](#_ENREF_8)].

**Programmatic aspects**

Meningococcal vaccination strategies in specific countries evolves in response to shifting epidemiology at a local/national level (in terms of risk in different age-groups and predominant serogroups), and the introduction and availability of newer vaccines. Most countries have a single national policy, although in some countries, this is devolved on a regional/state basis, implementing broader or more restricted routine use [[9-16](#_ENREF_9)].

Most countries utilize infant, toddler and adolescent immunisation, which varies widely across countries (comprehensively reviewed by Taha et al. [[17](#_ENREF_17)].) See Supplementary Fig. 2 below. In Europe, infant and toddler immunization is focused on MenC and MenB vaccination, and then adolescent MenACWY immunization [[9-11](#_ENREF_9)]. Some countries e.g., the Netherlands use quadrivalent vaccines for both toddlers and adolescents (and also in some Italian regions), while both France and Germany use MenC rather than MenACWY for routine infant and adolescent immunization [[9](#_ENREF_9),[10](#_ENREF_10)]. There are no infant/toddler recommendations in the US; routine adolescent quadrivalent immunization is recommended (at 11–12 years), along with MenB vaccines (under shared clinical decision-making) at 16–23 years [[13](#_ENREF_13)]. Canada also uses adolescent MenACWY immunization (except Manitoba, Quebec and Nunavut, where MenC booster is used).[[12](#_ENREF_12)] In Australia, MenACWY is given to toddlers and adolescents[[18](#_ENREF_18)]. MenB is also offered to at-risk ethnic groups (Aboriginal and Torres Strait Islander children) and for all infants and adolescents across South Australia State [[11](#_ENREF_11)]. Infant MenC and adolescent MenACWY immunization is used in Brazil [[14](#_ENREF_14)], while in Chile, infant MenACWY immunization is in use (introduced to address the dramatic increase in disease due to serogroup W) [[19](#_ENREF_19),[15](#_ENREF_15)]. In Saudi Arabia, conjugate MenACWY is long-established in the childhood immunization programme, with more recent introduction for adolescents. Other countries within the Gulf Cooperation Council (GCC) region also employ infant/toddler MenACWY (Kuwait, Bahrain) while the United Arab Emirates uses adolescent immunization [[20](#_ENREF_20)]. None of these GCC countries utilize MenB in their national program [[20](#_ENREF_20)].

**Supplementary Fig. 2** Meningococcal vaccines in national immunization programmes in selected countries [[17](#_ENREF_17)]

**
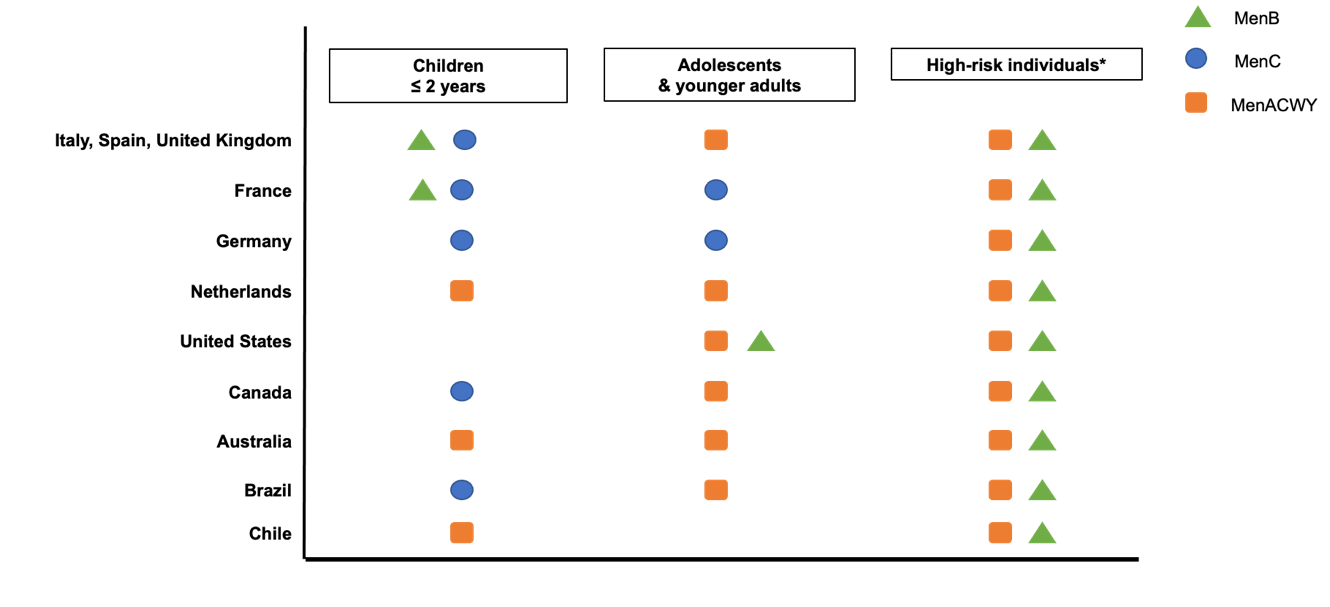
**

In some countries (e.g., Australia, Canada) regional policy may differ from national policy. *High-risk individuals include congenital or acquired immunosuppressive conditions (asplenia/splenic dysfunction sickle cell disease, complement deficiency) or receiving specific immunosuppressive medications (e.g., complement component inhibitors), people living with HIV and AIDS (PLWHA), specific groups such as those living in crowded living accommodation, and men who have sex with men (MSM). Specific indications vary in different countries.

**IMD incidence**

Incidence rates are far higher in infants and younger children than observed in older age-groups, highlighting the importance of childhood and adolescent immunization [[1](#_ENREF_1),[2](#_ENREF_2)]. While rates have declined substantially over the past decade, surveillance data for Europe (sourced from the European Centre for Disease Prevention and Control [ECDC]) indicates that in 2019 the IMD notification rate in infants <1 year was 7.4 per 100,000, 2.0 per 100,000 in those aged 1–4 years, with far lower incidence in 15–24 year olds (0.9 per 100,000) and those ≥50 years (0.5 per 100,000) [[21](#_ENREF_21)]. While rates are lower in the US [[22](#_ENREF_22)], similar patterns are seen (Supplementary Fig. 3).

**Supplementary Fig. 3** IMD incidence rates per 100,000 rates across Europe (EU/EEA) and the United States stratified by age. European data sourced from the European Centre for Disease Prevention and Control (ECDC) Surveillance Atlas of Infectious Diseases tool [[21](#_ENREF_21)]. Data for the United States as reported by the Centers for Disease Control and Prevention (CDC) [[22](#_ENREF_22)]


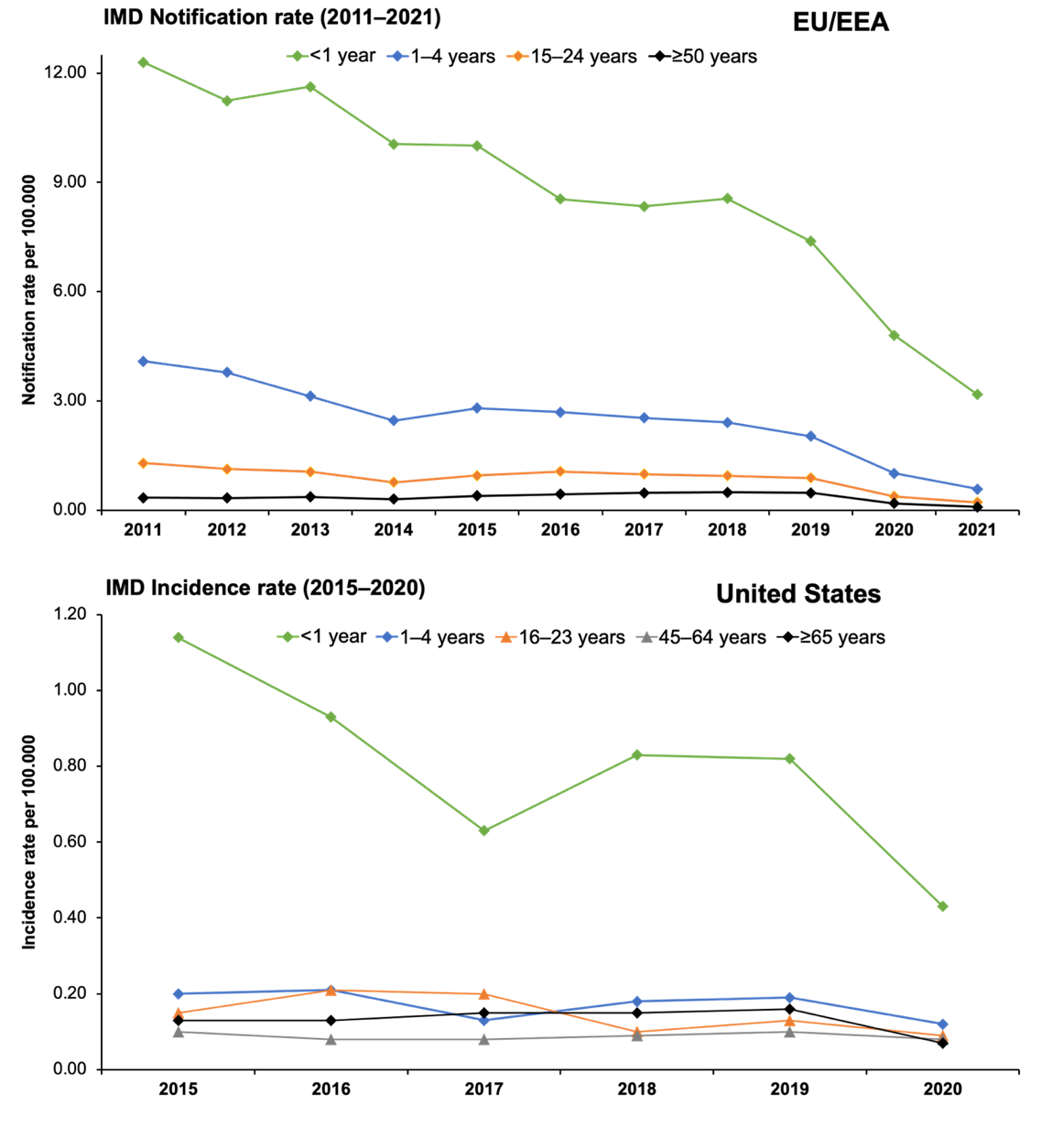


**Supplementary Fig. 4** Distribution of invasive meningococcal disease case burden in select age-strata; United Kingdom, France. Data sourced from the European Centre for Disease Prevention and Control (ECDC) Surveillance Atlas of Infectious Diseases tool [[21](#_ENREF_21)]. Data for the United Kingdom are not available for 2020/2021


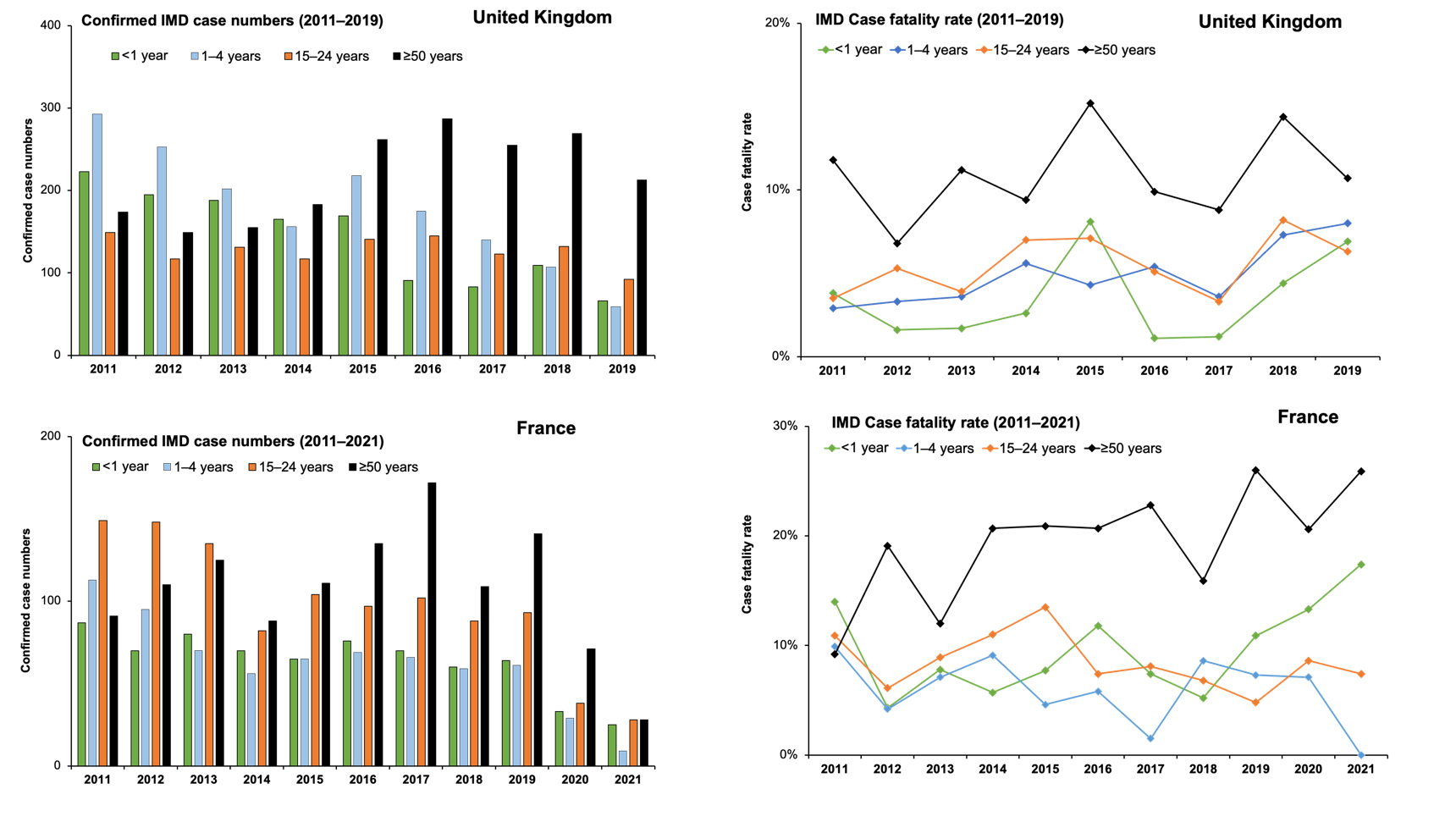


**Supplementary Fig. 5** Case fatality rates in select age-strata; United Kingdom, France. Data sourced from the European Centre for Disease Prevention and Control (ECDC) Surveillance Atlas of Infectious Diseases tool [[21](#_ENREF_21)]. Data for the United Kingdom are not available for 2020/2021


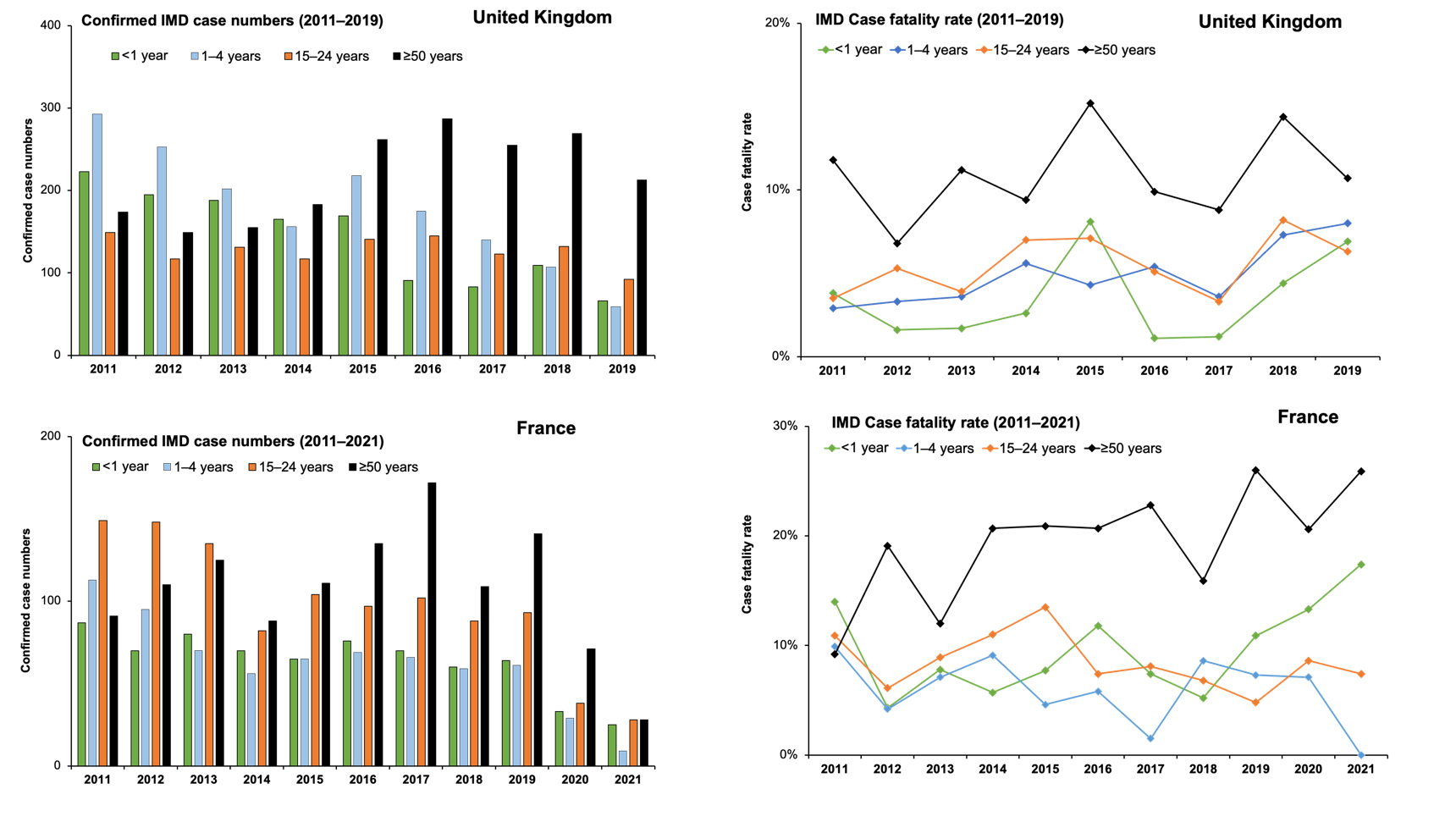


# **References for Supplementary Material**

1. Parikh SR, Campbell H, Bettinger JA, Harrison LH, Marshall HS, Martinon-Torres F et al (2020) The everchanging epidemiology of meningococcal disease worldwide and the potential for prevention through vaccination. J Infect 81(4):483-498. <https://doi.org/10.1016/j.jinf.2020.05.07910.1016/j.jinf.2020.05.079>.

2. Borrow R, Alarcon P, Carlos J, Caugant DA, Christensen H, Debbag R et al (2017) The Global Meningococcal Initiative: global epidemiology, the impact of vaccines on meningococcal disease and the importance of herd protection. Expert Rev Vaccines 16(4):313-328. <https://doi.org/10.1080/14760584.2017.125830810.1080/14760584.2017.1258308>.

3. Clark SA, Borrow R (2020) Herd Protection against Meningococcal Disease through Vaccination. Microorganisms 8(11):1675. <https://doi.org/10.3390/microorganisms811167510.3390/microorganisms8111675>.

4. McMillan M, Chandrakumar A, Wang HLR, Clarke M, Sullivan TR, Andrews RM et al (2021) Effectiveness of Meningococcal Vaccines at Reducing Invasive Meningococcal Disease and Pharyngeal Neisseria meningitidis Carriage: A Systematic Review and Meta-analysis. Clin Infect Dis 73(3):e609-e619. <https://doi.org/10.1093/cid/ciaa173310.1093/cid/ciaa1733>.

5. Mbaeyi S, Sampo E, Dinanibe K, Yameogo I, Congo-Ouedraogo M, Tamboura M et al (2020) Meningococcal carriage 7 years after introduction of a serogroup A meningococcal conjugate vaccine in Burkina Faso: results from four cross-sectional carriage surveys. Lancet Infect Dis 20(12):1418-1425. <https://doi.org/10.1016/S1473-3099(20)30239-510.1016/S1473-3099(20)30239-5>.

6. Carr JP, MacLennan JM, Plested E, Bratcher HB, Harrison OB, Aley PK et al (2022) Impact of meningococcal ACWY conjugate vaccines on pharyngeal carriage in adolescents: evidence for herd protection from the UK MenACWY programme. Clin Microbiol Infect 28(12):1649 e1641-1649 e1648. <https://doi.org/10.1016/j.cmi.2022.07.00410.1016/j.cmi.2022.07.004>.

7. Campbell H, Andrews N, Parikh SR, White J, Edelstein M, Bai X et al (2022) Impact of an adolescent meningococcal ACWY immunisation programme to control a national outbreak of group W meningococcal disease in England: a national surveillance and modelling study. Lancet Child Adolesc Health 6(2):96-105. <https://doi.org/10.1016/S2352-4642(21)00335-710.1016/S2352-4642(21)00335-7>.

8. Yezli S, Bin Saeed AA, Assiri AM, Alhakeem RF, Yunus MA, Turkistani AM et al (2016) Prevention of meningococcal disease during the Hajj and Umrah mass gatherings: past and current measures and future prospects. Int J Infect Dis 47:71-78. <https://doi.org/10.1016/j.ijid.2015.12.01010.1016/j.ijid.2015.12.010>.

9. Martinon-Torres F, Taha MK, Knuf M, Abbing-Karahagopian V, Pellegrini M, Bekkat-Berkani R et al (2022) Evolving strategies for meningococcal vaccination in Europe: Overview and key determinants for current and future considerations. Pathog Glob Health 116(2):85-98. <https://doi.org/10.1080/20477724.2021.197266310.1080/20477724.2021.1972663>.

10. Pinto Cardoso G, Lagree-Chastan M, Caseris M, Gaudelus J, Haas H, Leroy JP et al (2022) Overview of meningococcal epidemiology and national immunization programs in children and adolescents in 8 Western European countries. Front Pediatr 10:1000657. <https://doi.org/10.3389/fped.2022.100065710.3389/fped.2022.1000657>.

11. Sohn WY, Tahrat H, Novy P, Bekkat-Berkani R (2022) Real-world implementation of 4-component meningococcal serogroup B vaccine (4CMenB): implications for clinical practices. Expert Rev Vaccines 21(3):325-335. <https://doi.org/10.1080/14760584.2022.202188110.1080/14760584.2022.2021881>.

12. Robinson JL (2018) Update on invasive meningococcal vaccination for Canadian children and youth. Paediatr Child Health 23(1):e1-e4. <https://doi.org/10.1093/pch/pxx16210.1093/pch/pxx162>.

13. Mbaeyi SA, Bozio CH, Duffy J, Rubin LG, Hariri S, Stephens DS et al (2020) Meningococcal Vaccination: Recommendations of the Advisory Committee on Immunization Practices, United States, 2020. MMWR Recomm Rep 69(9):1-41. <https://doi.org/10.15585/mmwr.rr6909a110.15585/mmwr.rr6909a1>.

14. Aparecido Nunes A, De Jesus Lopes De Abreu A, Cintra O, M ACTC, Barbosa Coelho E, Nogueira Castro De Barros E (2021) Meningococcal disease epidemiology in Brazil (2005-2018) and impact of MenC vaccination. Vaccine 39(3):605-616. <https://doi.org/10.1016/j.vaccine.2020.11.06710.1016/j.vaccine.2020.11.067>.

15. Villena R, Valenzuela MT, Bastias M, Santolaya ME (2022) Invasive meningococcal disease in Chile seven years after ACWY conjugate vaccine introduction. Vaccine 40(4):666-672. <https://doi.org/10.1016/j.vaccine.2021.11.07510.1016/j.vaccine.2021.11.075>.

16. Sulis G, Horn M, Borrow R, Basta NE (2022) A comparison of national vaccination policies to prevent serogroup B meningococcal disease. Vaccine 40(26):3647-3654. <https://doi.org/10.1016/j.vaccine.2022.04.10110.1016/j.vaccine.2022.04.101>.

17. Taha MK, Bekkat-Berkani R, Abitbol V (2023) Changing patterns of invasive meningococcal disease and future immunization strategies. Hum Vaccin Immunother 19(1):2186111. <https://doi.org/10.1080/21645515.2023.218611110.1080/21645515.2023.2186111>.

18. Sharma K, Chiu C, Wood N (2019) Meningococcal vaccines in Australia: a 2019 update. Aust Prescr 42(4):131-135. <https://doi.org/10.18773/austprescr.2019.04210.18773/austprescr.2019.042>.

19. Villena R, Valenzuela MT, Bastias M, Santolaya ME (2019) Meningococcal invasive disease by serogroup W and use of ACWY conjugate vaccines as control strategy in Chile. Vaccine 37(46):6915-6921. <https://doi.org/10.1016/j.vaccine.2019.09.05010.1016/j.vaccine.2019.09.050>.

20. Al Awaidy S, Ozudogru O, Badur S (2023) Meningococcal disease within the Gulf Cooperation Council Countries. Hum Vaccin Immunother 19(1):2193120. <https://doi.org/10.1080/21645515.2023.219312010.1080/21645515.2023.2193120>.

21. ECDC Surveillance atlas of infectious diseases. Stockholm: ECDC. <https://ecdc.europa.eu/en/surveillance-atlas-infectious-diseases> [accessed 12 May 2023].

22. US Centers for Disease Control and Prevention (CDC). Enhanced Meningococcal Disease Surveillance Reports, 2015–2020. <https://www.cdc.gov/meningococcal/surveillance/index.html> [accessed 11 May 2023].
